# Risks and errors in medicine. Concept and evaluation of an optional study module with integrated teaching of ethical, legal and communicative competencies

**DOI:** 10.3205/zma001177

**Published:** 2018-08-15

**Authors:** Jan Schildmann, Sabine Salloch, Tim Peters, Tanja Henking, Jochen Vollmann

**Affiliations:** 1 Martin-Luther-Universität Halle-Wittenberg, Institut für Geschichte und Ethik der Medizin, Halle, Germany; 2 Ruhr-Universität Bochum, Institut für Medizinische Ethik und Geschichte der Medizin, Bochum, Germany; 3 Universitätsmedizin Greifswald, Institut für Ethik und Geschichte der Medizin, Greifswald, Germany; 4 Ruhr-Universität Bochum, Zentrum für Medizinische Lehre, Bochum, Germany; 5 Hochschule für angewandte Wissenschaften Würzburg-Schweinfurt, Würzburg, Germany

**Keywords:** Error, Risks, Medical Ethics, Medical Law, Medical Studies, Evaluation

## Abstract

**Objective: **The presentation and discussion of the content, methodology and evaluation results of a course on “Risks and Errors in Medical Science”, adopting an integrated approach for the teaching of ethical, legal and communicative aspects.

**Method: **Written, structured evaluation using the adapted evaluation form “Evasys” as well as open questions on positive and negative aspects of the course and assessment of the effects thereof from the participants’ viewpoint. The free text responses are evaluated according to the principles of qualitative content analysis.

**Results: **Thirty-two from thirty-six participants (88.9%) evaluated the course in written form. The value for the global assessment of the course is a 1.7 (1=very good, 6=unsatisfactory). The self-assessed learning success was evaluated with a 1.9. In the qualitative analysis of the free text answers the case orientated teaching, the use of simulated patients as well as the legal content and the ethical models of the doctor-patient relationship were evaluated positively. Negative aspects of the course were, among other things, the weighting of the contents and the limited amount of time allotted. Impacts and changes were identified by the students in terms of knowledge of legal aspects, reflection on their own action and the training and further development of communication aptitudes. The students saw the relevance of the course for their studies especially in the supplement to the compulsory curriculum as well as in the significance of the content of medical law.

**Conclusions: **The experience of the authors and the written evaluation results show that the concept of integrated teaching can be implemented in medical studies and that it is predominantly evaluated positively. The primary challenges are the weighting of the different contents and methods as well as the comparatively high demand for coordination among representatives of various disciplines.

## Zusammenfassung

**Zielsetzung: **Darstellung und Diskussion der Inhalte, Methodik und Evaluationsergebnisse einer Lehrveranstaltung zu „Risiken und Fehler in der Medizin“ unter Verwendung eines integrierten Ansatzes zur Vermittlung ethischer, rechtlicher und kommunikativer Aspekte.

**Methodik: **Schriftliche strukturierte Evaluation mittels des adaptierten Evaluationsbogen „Evasys“ sowie ergänzender offener Fragen zu positiven und negativen Aspekten des Kurses und Einschätzung zu den Auswirkungen des Kurses aus Sicht der Teilnehmenden. Die Auswertung der Freitextantworten erfolgt nach Prinzipien der qualitativen Inhaltsanalyse.

**Ergebnisse: **32 der 36 Teilnehmenden (88,9%) evaluierten die Lehrveranstaltung schriftlich. Der Wert für die globale Beurteilung der Lehrveranstaltung liegt bei 1,7 (1=sehr gut, 6=ungenügend). Der selbst eingeschätzte Lernerfolg wurde mit 1,9 angegeben. Im Rahmen der qualitativen Analyse der Freitextantworten wurden die Fallorientierung, der Einsatz von Simulationspatienten sowie die juristischen Inhalte und die ethischen Modelle der Arzt-Patient-Beziehung positiv gewertet. Negative Aspekte des Kurses waren unter anderem die Gewichtung der Inhalte und der geringe zeitliche Umfang. Auswirkungen und Veränderungen wurden von den Studierenden in Bezug auf die Kenntnis rechtlicher Aspekte, die Reflexion auf das eigene Handeln sowie die Ausbildung und Weiterentwicklung kommunikativer Kompetenzen benannt. Die Bedeutung der Lehrveranstaltung für das Studium sahen die Studierenden unter anderem in der Ergänzung zum Pflichtcurriculum sowie der Bedeutung von Inhalten des Medizinrechts.

**Schlussfolgerungen: **Die Erfahrungen der Autoren und die schriftlichen Evaluationsergebnisse zeigen, dass das integrierte Lehrkonzept im Medizinstudium umsetzbar ist und überwiegend positiv bewertet wird. Wesentliche Herausforderungen bilden die Gewichtung der unterschiedlichen Inhalte und Methoden sowie der vergleichsweise hohe Abstimmungsbedarf der Vertreter der unterschiedlichen Fachrichtungen.

## Introduction

Ethical, legal and communicative teaching contents in Germany are subject to regulations governing medical licensure. To date, however, only a few courses are offered in which the teaching of ethical, legal and communicative aspects of the doctor-patient relationship take place as elements of such classes [1], [2]. Professional conduct in clinical practice often requires a combination of ethical, legal and communication competencies. It is not sufficient, for instance, for the physician to possess knowledge and analytic competence regarding law and ethics, but to be unable to implement the established normative principles of communication between physician and patient. Furthermore, typical ethical and legal challenges frequently arise in the doctor-patient relationship, which require identification and competent handling by the physician. As an example, what course of action should the doctor take in cases in which an underage patient suffering from cancer rejects further life-prolonging treatment measures? What legal consequences ought to be considered when a therapeutic error is presented to the patient? What form of risk reporting communication should be adopted to ensure the patients’ ability to make their own decisions? In addition to these and many other such examples drawn from clinical practice in which ethical, legal and communication challenges are closely intertwined, there are also relevant theoretical links between the three areas. One such example is the ethical and legal analyses of communication models, as in shared decision-making [3], [4], [5]. The instances mentioned above evince that integrated teaching of ethical, legal and communicative competencies is essential both regarding practical medical competence and in terms of theoretical aspects relating to “practice-oriented medicine”. To the best of our knowledge, however, there are only a few conceptual contributions or reports from teaching practice [1], [2] regarding the integrated instruction of these areas of medical training.

Hereafter, the integrated teaching of ethical, legal and communicative content in clinical medicine is presented and discussed with reference to the course “Risks and Errors in Medicine” (elective in the first and second study phase at the Medical Faculty of the Ruhr-Universität Bochum). In this paper, the term “integrated” communication implies two meanings. Firstly, the knowledge and methods of the three fields ethics, law and communication are combined with a view to an overriding goal – in this case, the students’ ability to deal professionally with risks and errors. Secondly, the term refers to the necessary and comprehensive coordination of content and methods between the three areas involved in the implementation of the course. The introduction to the subject matter is followed by a presentation of the course’s main learning objectives, contents and methods. Evaluation results are subsequently summarized with particular consideration paid to the qualitative analysis of the students’ free text answers on the subject of the integrated teaching concept. In the concluding session, opportunities and limitations of integrated teaching– also considering the practical challenges – will be discussed.

## Risks and Errors in Clinical Medicine. Introduction to the Content

Dealing with risks and errors is an important part of medical work [6], [7]. The common feature of both topics is that they are negatively connotated from the point of view of patients, relatives and the medical team. Errors and the manifestation of risks can challenge confidence in the “medical system.” Medicine depends on a high degree of trust in society and acceptance of immanent rules and procedures. When patients are harmed by the manifestation of risks or errors, this trust is questioned or even damaged. The essential difference between errors and risks is that the former is defined as an incident of avoidable harm, whereas risks refer to undesirable incidents, in other words, risks of harm that may become manifest despite error-free treatment. 

Medical errors and risks place considerable demands on doctors’ ethical, legal and communication competencies. The discussion of risks by doctors is either not carried out or else carried out incorrectly [8]. Knowledge of the ethical and legal principles of information of patients and competences to communicate risk are important prerequisites for an appropriate handling of risks in medicine. Regarding medical errors, shame, fear of legal consequences and feared disadvantages in professional advancement are common reasons for them being taboo subjects. Empirical studies show that a lack of, or nonprofessional communication of errors, both among the team and relating to the patient, may cause additional stress for patients and their relatives. Furthermore, the lack of communication about errors makes it more difficult to avoid errors in the future [6], [9]. 

In view of the considerable ethical, legal and communicative requirements in the professional handling of errors and risks as well as the lack of courses offered on these topics for medical students at the Ruhr University Bochum, the authors, for their part expert representatives in medicine, medical ethics, law and linguistics, have co-designed and conducted a series of courses on this issue.

## Project Outline

The course in the form presented in this article was offered for the first time in the summer semester of 2013 by the Institute of Medical Ethics and History of Medicine in cooperation with the Center for Medical Education (both at Ruhr University Bochum) as an elective subject for students of human medicine of all semesters. The number of participants is limited to a total of twelve students. Grading is based on a paper in which ethical, legal and communicative aspects of a concrete case study are analyzed.

**Figure 1:**
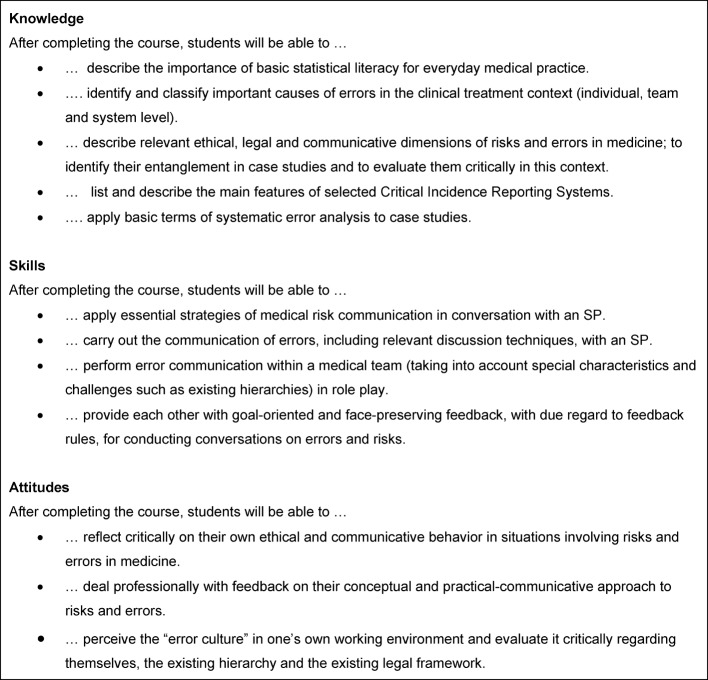
Learning objectives of the courses, summarized.

The seminar is divided into four sessions, the first two of which are devoted to the topic of “risks” while sessions 3 and 4 deal with the topic of “error”. Discussions with simulated patients (SP) will take place in both thematic blocks. A detailed presentation of the teaching content and methods used can be found in Table 1 (Tab. 1). A case study for each of the subject areas “Risks” and “Errors” is attached (see Attachment 1 and Attachment 2). The learning objectives are summarized in Figure 1 (Fig. 1).

## Methods of Evaluation

The evaluation by the students took place orally and in written form following the last session. The evaluation form “Evasys” (evaluation of content and didactic aspects of the course in school grades), which has been tested for many years in the faculty, was adapted and supplemented by open questions on positive and negative aspects of the course, as well as the impact on the individual and respectively assessment of the course for later work (see Attachment 3 for questionnaire). Due to the small number of students and the focus of the evaluation on feedback on the integrated teaching concept, emphasis is placed on the qualitative evaluation of the free text responses. The written feedback submitted by the students was evaluated according to the principles of qualitative content analysis [10]. Consequently, all free text answers

1. “What I liked about the course...”,
2. “What I didn’t like, or didn´t like so much about the course was....”,
3. “Did the course have any effect on you?” and
4. “Your assessment of the importance of the course for the studies”

from summer semester 2013 to winter semester 2014/2015 were summarized and divided into segments by the first author. In a next step, the quotations of three co-authors were, guided by rules and independently of each other, grouped into superordinate categories regarding content. Based on this, a category tree including all quotations was created by the first author. Finally, all authors involved in the evaluation discussed and consented to the results. In the following, we present the different categories and typical sample quotes from the students to illustrate each of the respective categories.

## Evaluation Results

Thirty-six students attended one of the four courses from summer semester 2013 to winter semester 2014/2015. Evaluation forms of thirty-two students (88.9%) were submitted. The value for the global assessment of the course is a 1.7 (1=very good, 6=unsatisfactory). The self-assessed learning success was evaluated as a 1.9. Selected categories formed on the basis of free text answers are presented below. Table 2 (Tab. 2), Table 3 (Tab. 3), Table 4 (Tab. 4) and Table 5 (Tab. 5) provide an overview of all categories and selected illustrative sample quotations.

In addition to general comments on **didactics and general conditions**, the *positive feedback* on the course (see Table 2 (Tab. 2)) related particularly **to** **case oriented teaching**, the use of **simulated patients** as well as the **legal content** and **ethical models of the doctor-patient relationship**. The **opportunity for discussion**, the** positive atmosphere** and the high performance of the** lecturers** were also rated positively.

*Negative aspects* of the course (see Table 3 (Tab. 3)) were the weighting of the content elements, particularly the lack of time allocated to the partially complex legal aspects. Regarding teaching methods, the large size of the group (max. 12 participants) in discussions with SPs was a topic of criticism. The late scheduling of the course (4.30-6.45 p.m.) was an object of specific criticism regarding course organization.

*Effects and changes* (see Table 4 (Tab. 4)) were identified by the students referring to their **knowledge of legal aspects, reflection on their own actions** and the training and further development of **communication competencies**. Additional feedback on the effects of the courses related to the impetus provided in the course for the **appropriate handling of risks and errors**.

The students saw the *importance of the course for their studies* (see Table 5 (Tab. 5)) primarily in the **supplement to the compulsory curriculum**, the** importance of law in medicine** and its **relevance for practice**, including** an improved communication** with patients.

## Discussion

The course on risks and errors deals with topics of specific importance for the medical profession [8], [11], [12] – topics which, to the best of the authors’ knowledge, have so far scarcely been dealt with within the framework of medical studies. A comparative study of the Nationalen Kompetenzbasierten Lernzielkatalog Medizin/National Competence-Based Learning Objectives Catalogue for Medicine [http://www.nklm.de] (cf. Table 6 (Tab. 6)) and the position paper of the Committee for Patient Safety and Error Management of the Gesellschaft für Medizinische Ausbildung [13] – published after the course was designed – shows that the course accounts for such key learning objectives and competences as are set out in the documents mentioned above. 

The experience of the authors after four semesters of joint development and implementation in addition to the written evaluation results of the students show that the treatment of the topic of risks and errors in medical studies can be implemented and is, for the most part, positively evaluated. The thematic focus “Risks and Errors” is cited in students’ feedback as an important and practice-oriented supplement to the compulsory curriculum. In this respect, our view of the need for teaching on these topics was confirmed. The integrated teaching of ethical, legal and communicative aspects is assessed positively by the students, at least indirectly. As outlined in the introduction, integration concerns both the orientation of the various contents and methods to the overall goal of enabling students to deal professionally with risks and mistakes and the detailed coordination between the courses’ three fields of ethics, law and communication. The overall positive feedback on the course may serve as empirical confirmation of the authors’ assessment that the approach adopted complements the traditional teaching in a significant way, at least from the perspective of the students participating. In view of the similar requirements for ethical, legal and communicative competences (e.g. information and consent, decisions at the end of life) also for other tasks in medical practice, the authors consider that the approach chosen of integrated teaching of competences in the fields of ethics, law and communication could be meaningfully extended to other topics. Such an investigation would cover both the conceptual basis of an integrated teaching of ethical, legal and communicative competencies outlined in this project report as well as the possibilities of transferring them to other topics.

The combination of the analysis of ethical models for the doctor-patient relationship, on the one hand, and the practical exercises with simulated patients, on the other, were, in the authors’ opinion, particularly helpful for the integration of ethical and communicative aspects. Based on the conceptual delimitation of a paternalistic, deliberative, interpretative or informative doctor-patient relationship [14], students acquire relevant knowledge regarding the ethical dimension of doctor-patient communication. Accordingly, focus in the discussion exercises is placed on the conscious and well-founded selection of one of the models of decision-making. This approach of critical reflection on the ethical dimension of communication differs from approaches to training communicative competences, which are concerned with the implementation of competencies previously definied as “good” [5], [15], [16].

The evaluation of the students also makes it clear that legal aspects of medical action in everyday clinical practice represent a desideratum in medical education. This coincides with students’ experiences and feedback from courses on clinical and ethical issues at the end of life in which the inclusion of legal content was requested by the students [17], [18]. In this context, it should be emphasized that the content learned predominantly in the field of forensic medicine, such as autopsy or medical forensic aspects, covers something other than the legal aspects of the doctor-patient-relationship taught by the authors in the course. According to the authors’ knowledge, the latter has yet to be systematically taught as an element of medical training. At the same time, from the outset basic legal knowledge represents an essential prerequisite for competent action in medical work.

The distribution and weighting of the various contents and methods represent key challenges in the implementation of the course. The responses of individual students show that there are differing expectations about the weighting of the three aspects ethics, law and communication. The selection and weighting of the subject matter within the framework of the course was also a recurring topic in the authors’ regular course discussions. It became clear during the course that the implementation of the interdisciplinary course depends on structural parameters and the individual commitment of the lecturers. On a structural level, the interdisciplinary Institute for Medical Ethics and History of Medicine and the existence of a SP pool and program with a large selection of well-trained SPs constituted important prerequisites. On an individual level, it should be noted that all the lecturers involved were required to make far greater than average efforts for the comprehensive content and methodological coordination than were demanded in other courses. Finally, on a critical note, it must be borne in mind that it is not possible to expand the course for all students in the teaching format presented here, at least not with the resources currently available.

### Methodological Strengths and Limitations

The project report is based on the experiences of four elective courses and written comments from 32 participating students. The qualitative methodology chosen facilitates a detailed understanding of the perceptions and evaluation of the course from the students’ perspective. Thus, the existing assumptions on the part of the lecturers regarding the value of the methodological approach chosen for the integrated communication of ethical, legal and communicative content and its practicability could be compared with the students’ perspective. In the light of the small number of responses from a selected cohort of students with above average motivation, we assume that these evaluation results cannot be applied to all students (limited representation). Furthermore, the qualitative analysis of free text answers as an explorative approach always represents an interpretative act by the evaluators, so that despite efforts to achieve transparency and traceability in the evaluation steps, subjective definitions are part of the evaluation as presented here.

## Competing interests

The authors declare that they have no competing interests. 

## Supplementary Material

case study for each of the subject area “Risks”

case study for each of the subject area “Errors”

questionnaire

## Figures and Tables

**Table 1:**
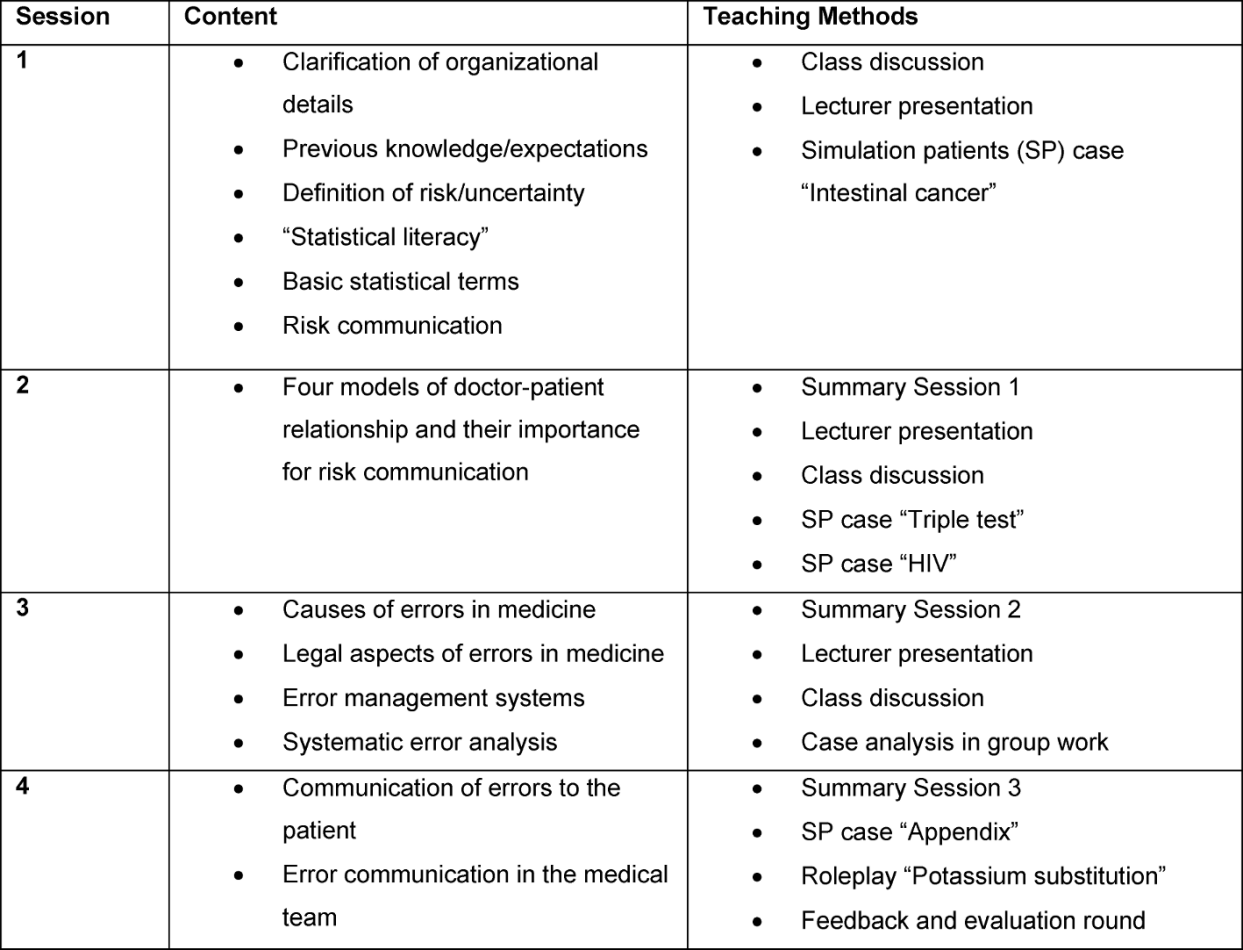
Content and didactic design of the seminar sessions

**Table 2:**
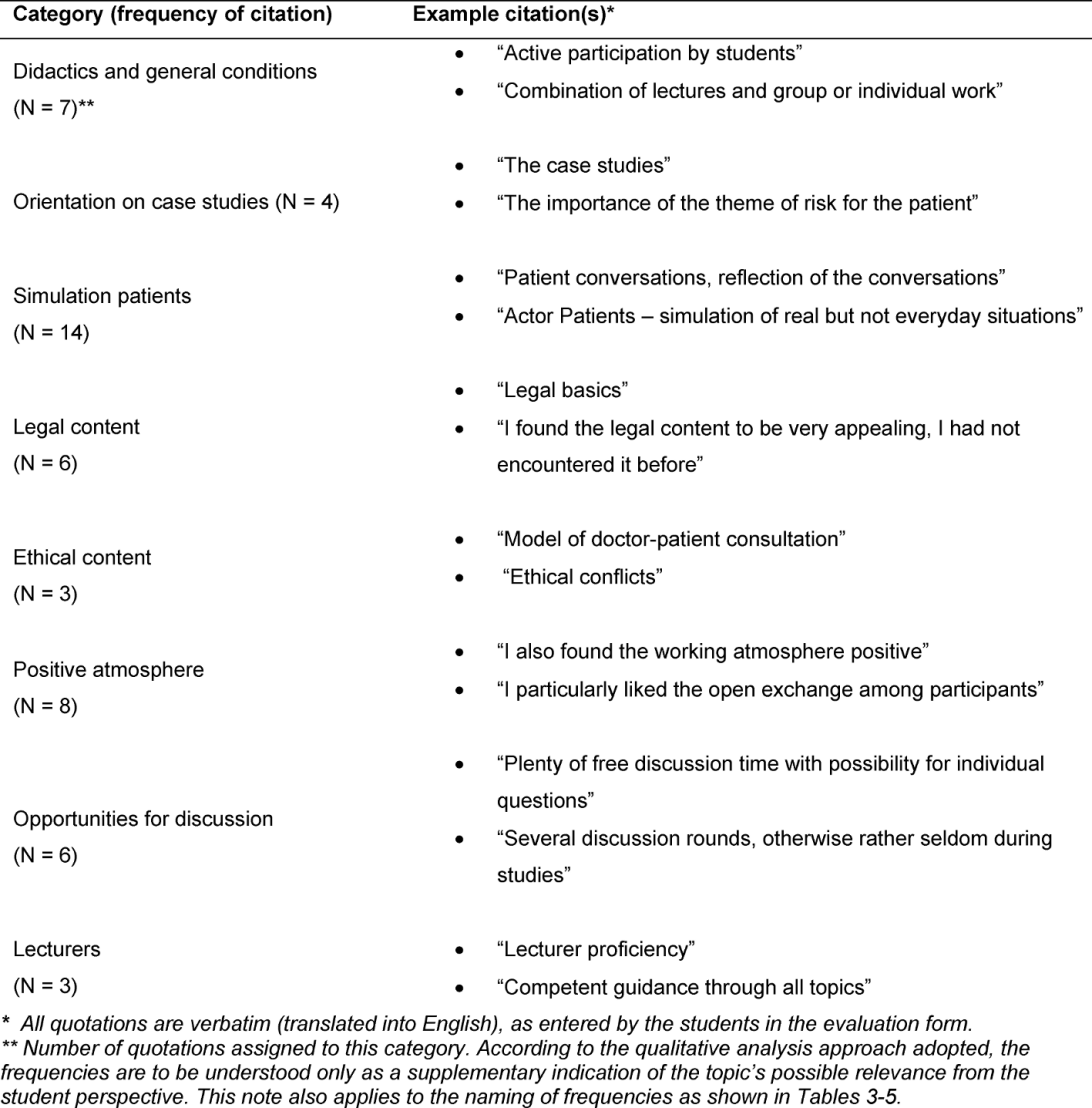
Positive feedback on the course

**Table 3:**
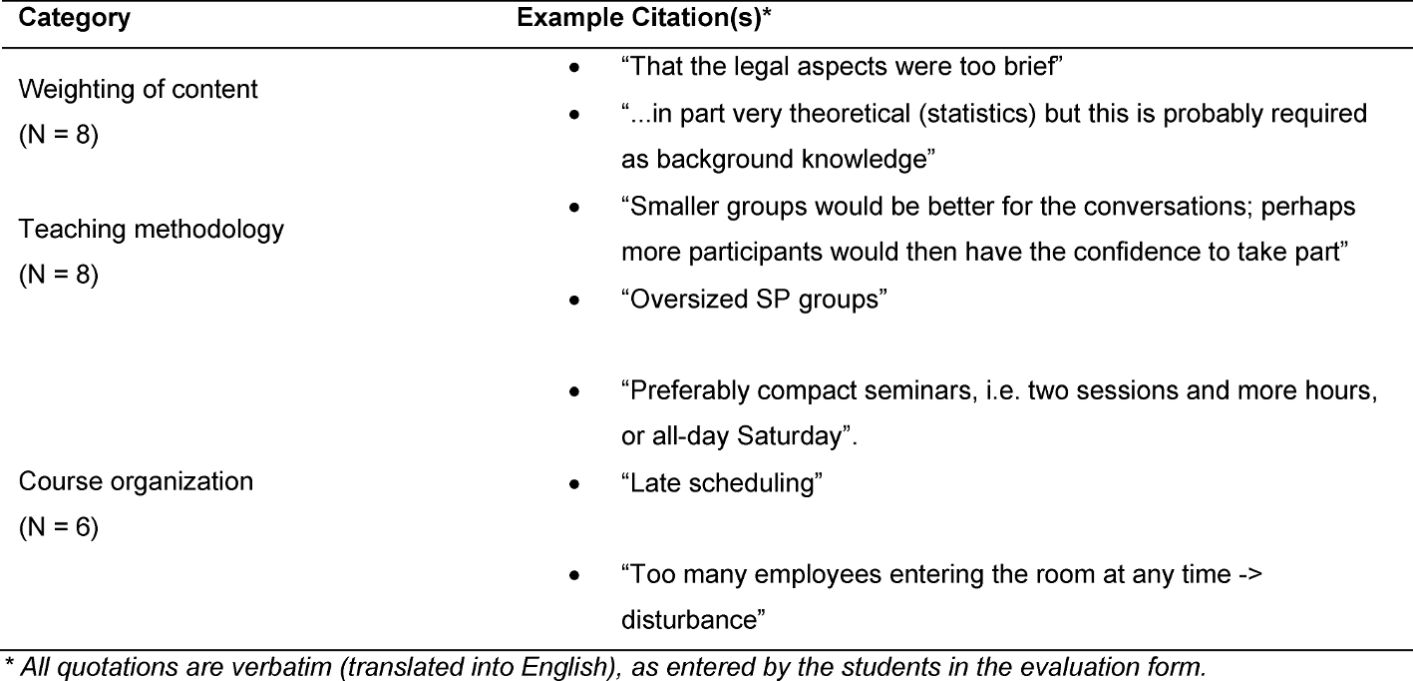
Negative aspects of the course

**Table 4:**
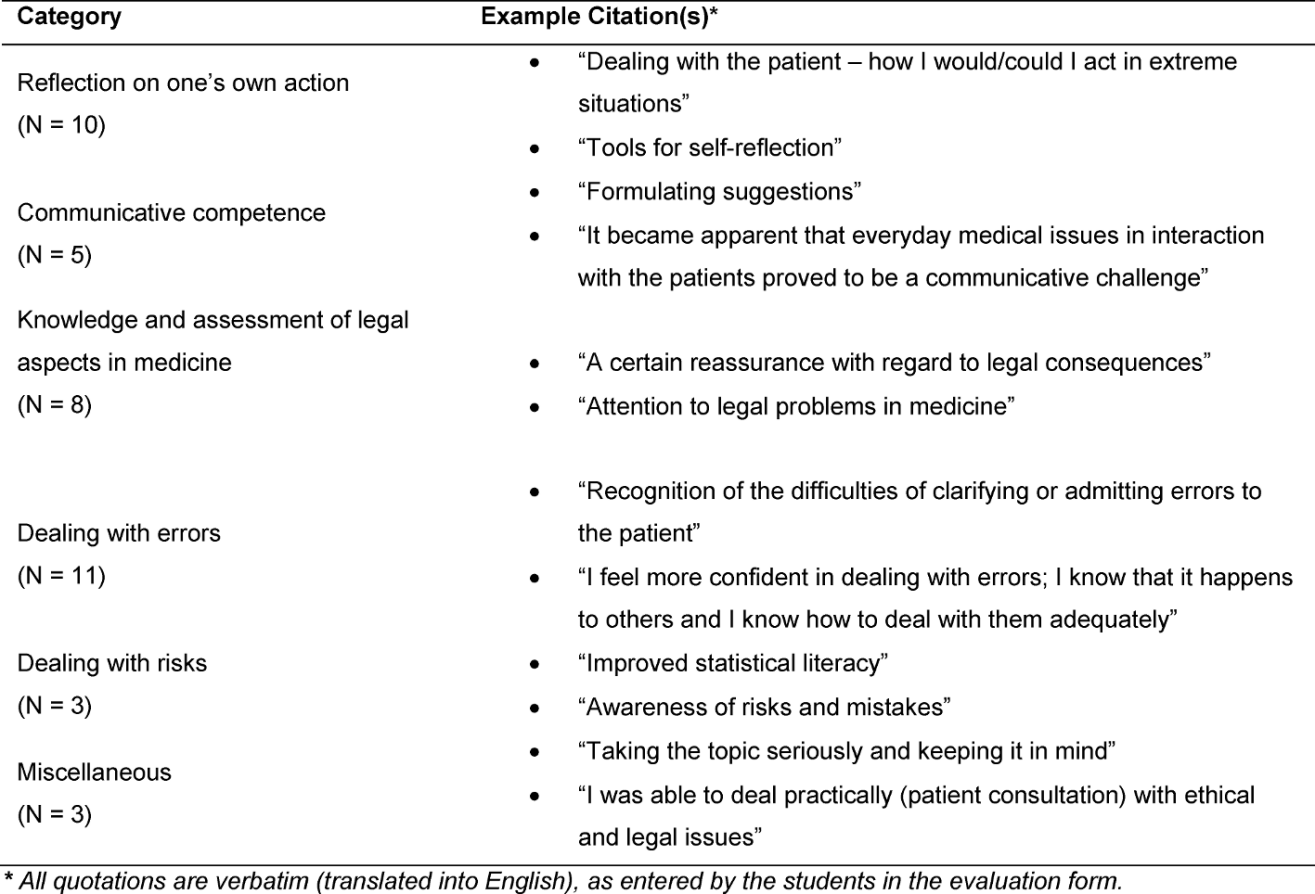
Effects and changes

**Table 5:**
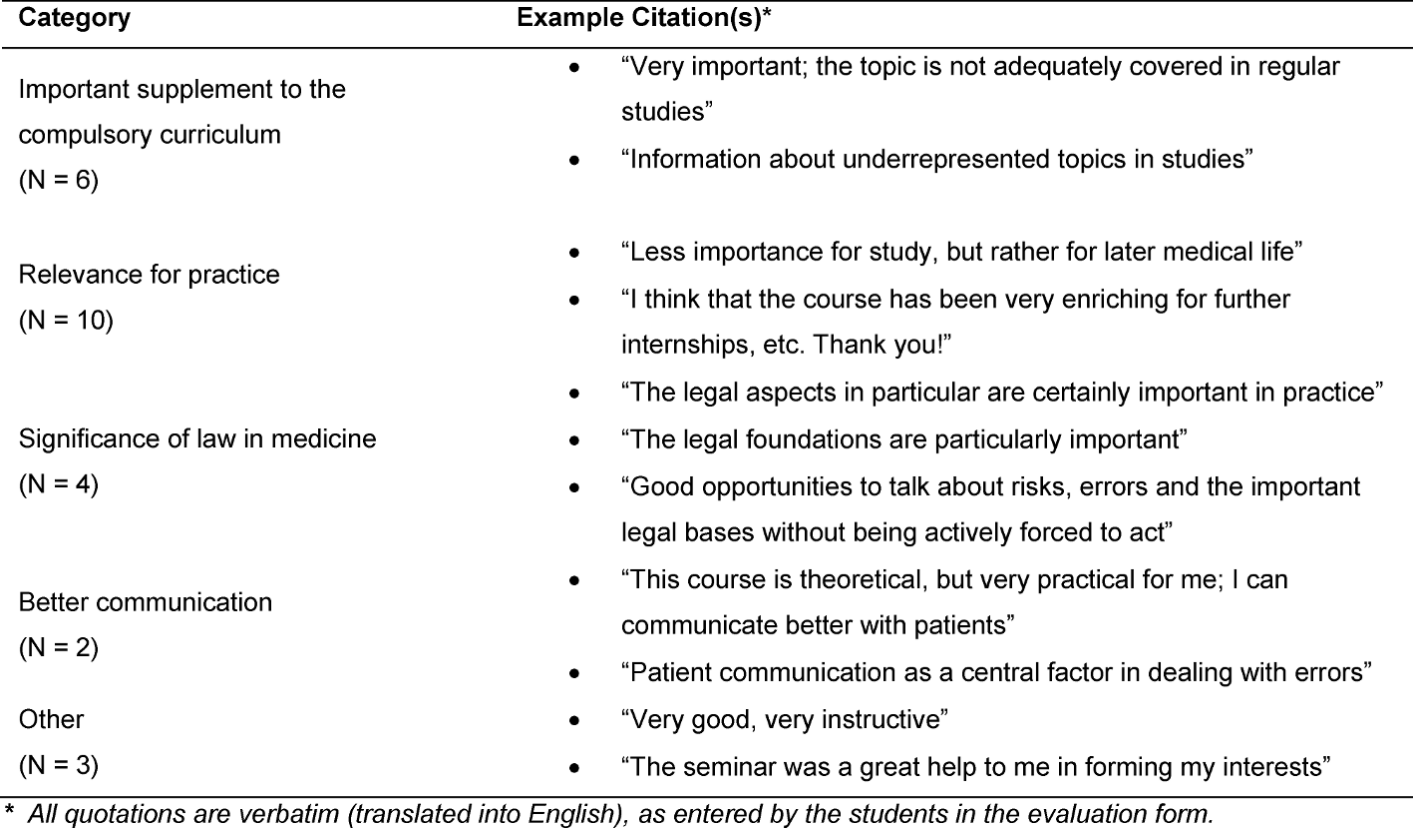
Importance of the course for their studies

**Table 6:**
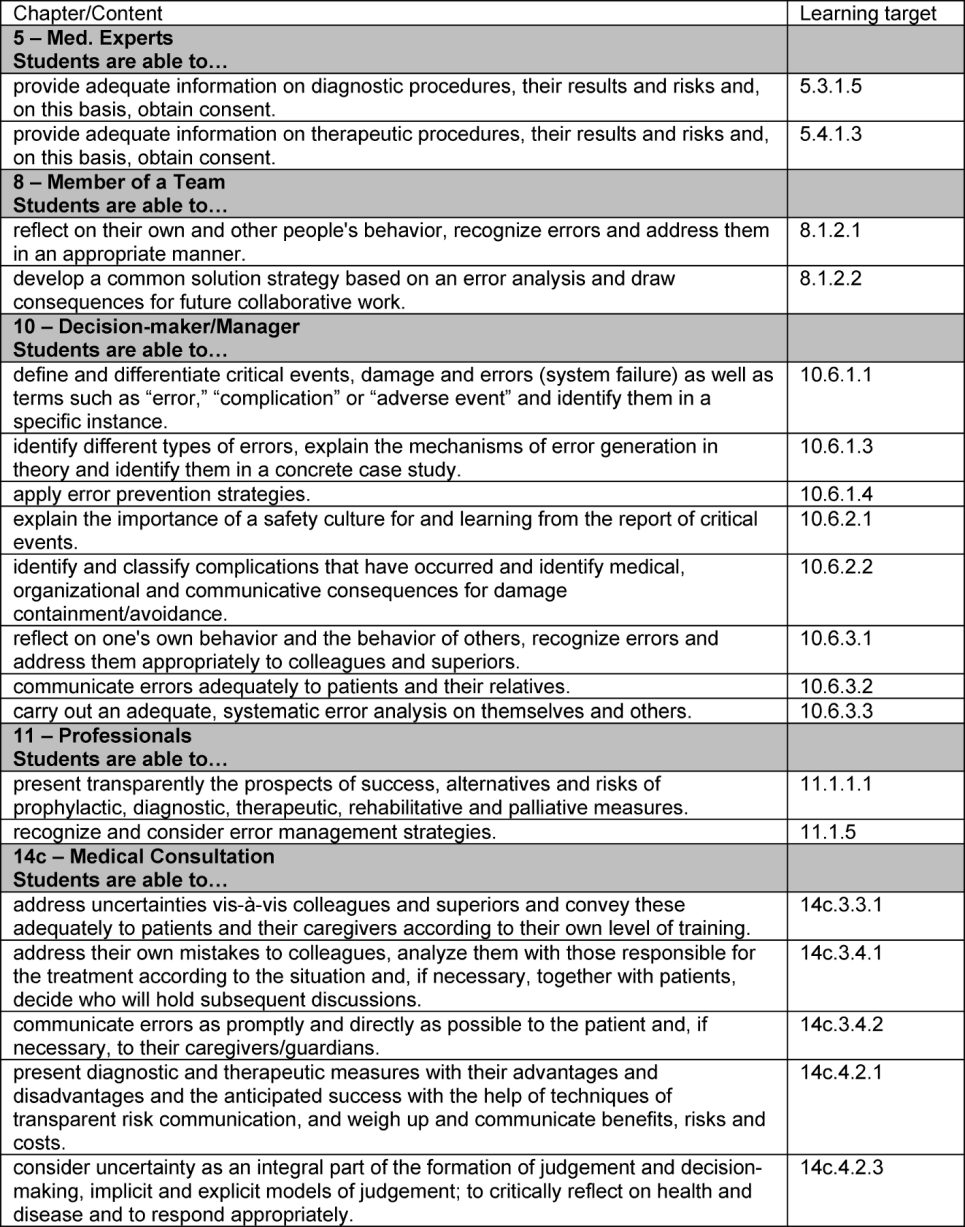
Nationaler Kompetenzbasierter Lernzielkatalog Medizin (selection of teaching aims relevant to the optional study module)
