# Supplementary material for: Risks and errors in medicine. Concept and evaluation of an optional study module with integrated teaching of ethical, legal and communicative competencies
Source: GMS J Med Educ. 2018 Aug 15;35(3):Doc31. doi: 10.3205/zma001177 (PMC6120147; doi:10.3205/zma001177)
Supplement: case study for each of the subject area “Risks” [file JME-35-31-s-001.pdf]

Intestinal Cancer

Discussion partner: Ms. X

Location: City Hospital, Surgery

You are treating a patient with colorectal cancer (UICC stage III). The patient has survived the operation (hemicolectomy) well and is recovering in the ward. Outpatient chemotherapy is still planned at intervals of several weeks. The patient was informed about everything but has now asked you once again to talk about the course of the disease and particularly the prognosis.

UICC Classification of Colon Carcinoma

The UICC classification of colon carcinoma is based on statistical studies that show, for example, that the prognosis of the disease deteriorates above a certain size of a tumor. The classification of a tumor disease, therefore, allows for prognostic statements and often determines further therapy.

UICC classification	
Stage Ia	Limitation of tumor infiltration to the mucosa and Tela submucosa (Dukes A)
Stage Ib	Limitation of tumor infiltration up to the tunica muscularis propria (Dukes A)
Stage II	Infiltration of subserosa or neighboring organs without lymph node metastasis (Dukes B)
Stage III	Lymph node metastasis (Dukes C)
Stage IV	Remote metastases (Dukes D)

Prognosis

The prognosis depends on the depth of infiltration into the intestinal wall and the presence of lymph node and distant metastases; the five-year survival rate is approximately 40 to 60 % on average. The most common finding is UICC stage III.

Five-year survival rate after UICC stage	
Stage I	approx. 80-100 %
Stage II	approx. 60-80 %
Stage III	approx. 30-60 %
Stage IV	approx. 0-57 %
