# Supplementary material for: Risks and errors in medicine. Concept and evaluation of an optional study module with integrated teaching of ethical, legal and communicative competencies
Source: GMS J Med Educ. 2018 Aug 15;35(3):Doc31. doi: 10.3205/zma001177 (PMC6120147; doi:10.3205/zma001177)
Supplement: case study for each of the subject area “Errors” [file JME-35-31-s-002.pdf]

Potassium Substitution

You work as a ward physician in an internal medical ward, where you currently have to treat over 20 patients with different indications. Although you have three years professional experience, in view of the large number of patients, you feel on the verge of being overwhelmed. For three weeks you have been supported by an assistant who will relieve you of some bureaucratic tasks and the taking of blood samples.

It is late in the morning and the laboratory results for the morning blood samples have just arrived. Since you are currently busy with other work, you give the laboratory note to the clerk so that he/she can “check it over.”

This is the starting point of the seminar discussion. To prepare yourself, however, here is some information on the further course of the discussion.

The assistant has found conspicuous potassium levels in two patients sharing the same room. He/she suspects that there may have been a switch of dosages, so that one patient would have received the potassium preparation intended for the other patient. The prescriptions are always made during the rounds.
At first you reject this “accusation” and find it inconceivable that you could be responsible for such an error. However, on closer inspection of the values, it seems to be the most probable cause.
Together with the intern, you consider how such mistakes can be avoided in the future. You continue to discuss whether the two patients concerned should be informed about the error.

Background Information:
***Potassium** belongs to the electrolytes (blood salts) which must be present in the blood in a certain concentration and are essential for the body's function. The normal value for potassium is 3.5 to 5.0 mmol/l. Both too high and too low potassium levels can have serious health consequences (nausea, muscle twitching, cardiac arrhythmia, even cardiac arrest).*
The potassium balance can be disturbed by various diseases, but also by medication. If potassium levels are too low, it must be supplied in tablet, effervescent or infusion form. If potassium levels are too high, it must be eliminated from the body, for example, by administering certain drugs, infusions or enemas.
