# Supplementary material for: Risks and errors in medicine. Concept and evaluation of an optional study module with integrated teaching of ethical, legal and communicative competencies
Source: GMS J Med Educ. 2018 Aug 15;35(3):Doc31. doi: 10.3205/zma001177 (PMC6120147; doi:10.3205/zma001177)
Supplement: questionnaire [file JME-35-31-s-003.pdf]

Evaluation Form

Session:	**Elective "Risks and Errors," WS 2013/14**
Location:	**Institute for Medical Ethics and History of Medicine, Ruhr-University Bochum**

Assessments on a value scale of 1 - 6

(1 = very good; 6 = insufficient**)**

How intensively did you prepare yourself for the seminar sessions?	
How did you feel about the working atmosphere?	
How was your active participation encouraged?	
How were students' questions answered?	
How do you assess the overall learning success of the seminar?	
How do you assess the performance of the seminar lecturers?	
**How would you rate the event overall?**	

Evaluation Form

What I particularly liked about the course:

What I did not like or what I liked less about the course:

Did the course have any effect on you? In what way?

What is your assessment of the importance of the seminar for your studies?
